# Integrative landscape of dysregulated signaling pathways of clinically distinct pancreatic cancer subtypes

**DOI:** 10.18632/oncotarget.25632

**Published:** 2018-06-26

**Authors:** Musalula Sinkala, Nicola Mulder, Darren Patrick Martin

**Affiliations:** ^1^ University of Cape Town, School of Health Sciences, Department of Integrative Biomedical Sciences, Computational Biology Division, Observatory, 7925, South Africa

**Keywords:** pancreatic cancer, bioinformatics, signal transduction pathways, integrative analysis, network analysis

## Abstract

Despite modern therapeutic advances, the survival prospects of pancreatic cancer patients have remained poor. Besides being highly metastatic, pancreatic cancer is challenging to treat because it is caused by a heterogeneous array of somatic mutations that impact a variety of signaling pathways and cellular regulatory systems. Here we use publicly available transcriptomic, copy number alteration and mutation profiling datasets from pancreatic cancer patients together with data on disease outcomes to show that the three major pancreatic cancer subtypes each display distinctive aberrations in cell signaling and metabolic pathways. Importantly, patients afflicted with these different pancreatic cancer subtypes also exhibit distinctive survival profiles. Within these patients, we find that dysregulation of the phosphoinositide 3-kinase and mitogen-activated protein kinase pathways, and p53 mediated disruptions of cell cycle processes are apparently drivers of disease. Further, we identify for the first time the molecular perturbations of signalling networks that are likely the primary causes of oncogenesis in each of the three pancreatic cancer subtypes.

## INTRODUCTION

Pancreatic cancer is the most lethal form of cancer. It has an extremely poor prognosis with less than 20% of patients surviving for more than one year following diagnosis [1, 2]. Factors contributing to reduced survival rates are the difficulty of diagnosing the disease during its early stages, the rapid progression of tumours with few specific associated symptoms, and the diversity of responses that different forms of pancreatic cancer have to anticancer drugs [3, 4]. Despite progress having been made towards understanding the histological phenotypes and molecular mechanisms at play, positive responses to conventional chemotherapy regimens have remained infrequent, and the overall survival rates of patients have not substantially improved [5].

A significant challenge to achieving better treatment outcomes has been the heterogeneity of pancreatic cancers. Underlying this heterogeneity is the vast array of somatic mutations that are acquired during oncogenesis, and the varied effects that these mutations have on cell signalling pathways [6, 7]. Recent analyses of genomic sequence datasets from patients with advanced disease have identified potential activating mutations, many of which occur in genes encoding proteins that might be suitable drug targets [1, 8]. In this regard the discovery of mutation hot-spots in various signalling kinases has already prompted the development of highly selective kinase inhibitors that are capable of specifically killing pancreatic cancer cells. Although the antitumor activities of some of these kinase inhibitors have been strong, they have rarely been long-lasting, with the targeted cancers frequently developing resistance [7]. There is, therefore, a pressing need to identify additional potential drug targets amongst the dysregulated signalling and metabolic pathway components that differentiate pancreatic cancer subtypes. Used in conjunction with kinase-inhibitors, novel drugs targeting these pathway components could yield pancreatic cancer therapies with longer lasting effectiveness.

Aiding in the discovery of novel drug targets has been the use of next-generation sequencing based analytical methods that simultaneously identify mutations in sequences and quantify the expression of all the cellular genes that might have an impact on cancer progression. In this regard, the Cancer Genome Atlas (TCGA) project, has performed a systematic genomic, transcriptomic and proteomic characterisation of matched healthy and cancerous tissue samples from thousands of individuals afflicted with a variety of cancers [9]. This data, together with matched clinical information is publicly available and includes data for 185 pancreatic cancer patients. Combining data on transcription levels, gene mutations, copy number alteration, protein expression levels and clinical information, the intention of such datasets is to uncover causal relationships between specific genetic and/or cellular aberrations and the onset of disease [10].

Recent genomic studies using such datasets have both provided insights into the biological heterogeneity of pancreatic cancer and identified genomic aberrations that may be of therapeutic and prognostic value [1, 8, 10]. These studies have identified somatic mutations in the proto-oncogene *KRAS* as a hallmark of pancreatic cancers in that more than 90% of pancreatic cancer cases have mutations in this gene [8, 10, 11]. Several other mutations are also strongly associated with the onset of pancreatic cancers, including homozygous deletions in the *TP53*, *SMAD4*, and *CDKN2A* tumour suppressor genes [10, 11]. Alteration in the *KRAS*, *TP53*, *SMAD4* and *CDKN2A* are considered as the critical drivers of pancreatic tumorigenesis and altered signalling through KRAS and p53 is associated with varied treatment response to therapy and disease outcomes [11–13]. Nonetheless, as in other cancers, genetic alterations and variations in gene expression also occur in many other genes. Specifically, genes involved in the RB, beta-catenin, PI3K-Akt, and NOTCH pathways, commonly exhibit alterations that likely contributed to tumour development and progression [2, 8, 11]. Further, while tumours displaying KRAS-pathway alterations either alone or in combination with TP53 pathway alterations have a poor prognosis, it has been shown that tumours with more complex pathway disruptions tend to have even poorer outcomes [12, 14].

Notably, these studies have highlighted the alterations in key genes that function in these various signaling pathways. However, these studies have relied on smaller and/or less detailed datasets than those which are now available and have therefore failed to comprehensively define the specific signaling network perturbations that arise during different forms of pancreatic cancer. Here we explore the molecular characteristics of the three major pancreatic cancer subtypes and define the altered signaling pathways and subcellular process that, besides differentiating these subtypes and potentially being the underlying drivers of oncogenesis, also present a variety of potential prognostic biomarkers and drug targets.

## RESULTS

We assembled a TCGA pancreatic ductal adenocarcinoma (PDAC) dataset comprising clinical information for 185 patients together with their associated cellular transcription data (based on RNA sequencing (RNA-Seq)), protein expression data (based on reverse phase protein array (RPPA)), and information on genomic mutations and copy number alterations (CNA). We performed, survival, clustering, and integrative pathway and network analyses of these diverse data types, both to classify the pancreatic cancers into different subtypes, and to reveal their clinical characteristics and the potential underlying causes of oncogenesis in of these different subtypes.

### Pancreatic cancer subtypes display distinctive clinical outcomes

**Figure 1:**
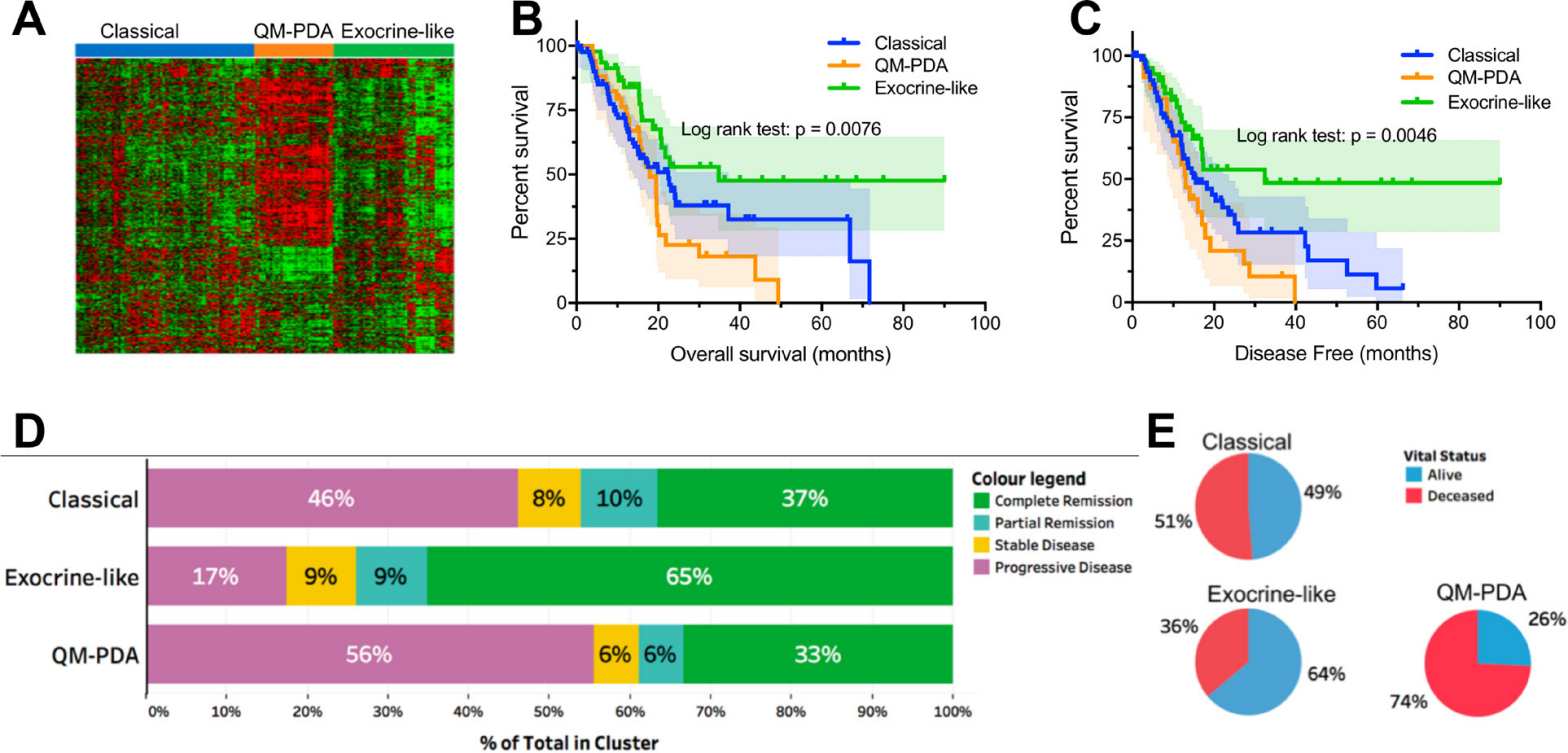
(**A**) Clustering of mRNA expression data identified three major pancreatic cancer subtypes, each with distinct expression patterns. (**B**) Kaplan–Meier Curves: overall patient survival periods were lower for patients with QM-PDAC and highest for those with EL-PDAC. Pairwise comparisons showed statistically significant differences between: C-PDAC vs. EL-PDAC (*χ*2 = 4.4, *p* = 0.036) and QM-PDAC vs. EL-PDAC (*χ*2 = 9.7, *p* = 0.002). (**C**) Kaplan–Meier Curves: disease-free survival months were lower for patients with QM-PDAC and highest for those with EL-PDAC: C-PDAC vs. EL-PDAC (χ2 = 5.3, *p* = 0.02) and QM-PDAC vs. EL PDAC (χ2 = 9.2, *p* = 0.002). (**D**) Vital statistics after the first course of treatment; only a quarter of QM-PDAC patients were alive compared with nearly half of C-PDAC patients and two-thirds of EL-PDAC patients. Odds ratios (95% CI): C-PDAC vs QM-PDAC = 2.7 (1.158–6.568), C-PDAC vs EL-PDAC = 0.541 (0.261–1.122), EL-PDAC vs QM-PDAC = 5.51(1.95–13.33). (**E**) Treatment outcomes after the first course of therapy were most favourable for EL-PDAC patients and least favourable for QM-PDAC patients.

Based on the mRNA transcription data we identified three major mRNA expression profiles using unsupervised hierarchical clustering (Figure 1A). Upon returning only exemplars for each profile, three PDAC subtypes were identified as: (1) quasi-mesenchymal PDAC (QM-PDAC; 35 samples), (2) classical PDAC (C-PDAC; 87 samples), and exocrine-like PDAC (EL-PDAC; 48 samples) [15, 17]. These three subtypes were associated with distinct overall survival, and duration of disease-free survival (Figure 1A and 1B). Specifically, both overall survival and the duration of disease-free survival were shorter for patients with the QM-PDAC subtype, intermediate for patients with the C-PDAC subtype and longer for patients with the EL-PDAC subtype. We observed a similar trend for treatment outcomes, with patients having QM-PDAC and EL-PDAC respectively displaying the worst and best outcomes (Figure 1C and 1D). Further, we did not observe significant associations between age, gender, or diabetes with the distribution of the PDAC subtypes (Supplementary Figure 1C).

### Gene expression and pathway characteristics of different PDAC subtypes

**Figure 2:**
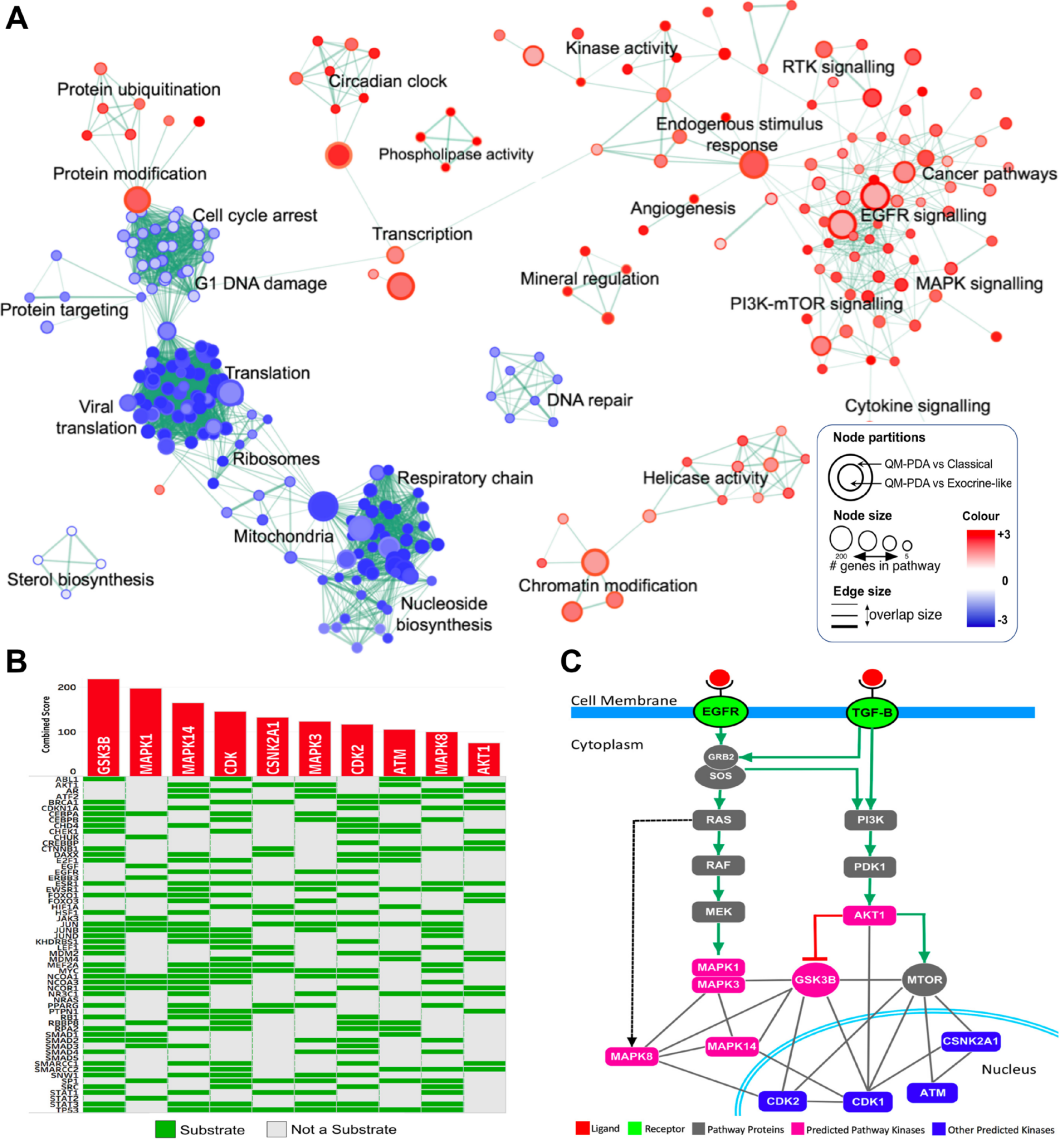
(**A**) Enrichment Map of QM-PDAC vs C-PDAC and EL-PDAC tumours: GSEA was used to obtain enriched gene ontology (GO)-terms that were visualised using the Enrichment Map plug-in for Cytoscape. Each node represents a GO-term with similar nodes clustered together and connected by edges with the number of known interactors between the nodes being represented by the thickness of edges. The size of each node denotes the gene set size for each specific node GO-term. A map comparing C-PDAC and EL-PDAC tumours is shown in Supplementary Figure 2. (**B**) Expression2Kinases solution: Heat map showing the top ten predicted kinases ranked according to their combined statistical score based on the number of substrates they phosphorylate within a protein-protein interaction subnetwork. Along the rows of the heatmap are proteins which are the substrates for kinases given along the columns of the heatmap. (**C**) Mapping of the top ten predicted kinases onto simplified models of the EGFR and TGF-Β signaling pathways. Six of the top ten ranked kinases (pink nodes) fall within these two pathways whereas the other predicted proteins are involved either directly in the cell cycle, or in the regulation of the cell cycle (blue nodes). We have provided simplified EGFR and TGF-β pathways with mapped mRNA expression levels in Supplementary Figure 5B.

We compared gene expression profiles between all pairs of PDAC subtypes and identified genes that were differentially expressed within the tumours of each PDAC subtype (see Supplementary File 1). Using gene set enrichment analysis (GSEA), we established that the genes which were differentially expressed between the subtypes were involved in a variety of different signalling pathways [16]. Compared with tumours of the other subtypes, those of the QM-PDAC subtype displayed elevated transcription levels for genes involved in the epidermal growth factor receptor (EGFR) signalling pathway, the transforming growth factor–beta (TGF-β) signalling pathway, the phosphoinositide 3-kinase-mechanistic target of rapamycin (PI3K-mTOR) oncogenic pathway, the mitogen-activated protein kinase (MAPK) oncogenic pathway and among others (Figure 2A). Dysregulation of the EGFR signalling pathway has previously been linked to tumour aggressiveness and reduced patient survival in various cancers including those of the breast and lung [17, 18]. The PI3K-mTOR pathway was inactive in EL-PDAC tumours but was activated in C-PDAC and QM-PDAC tumours. In other cancers, including those of the breast, gastrointestinal tract and prostate, activation of this pathway has been previously associated with significantly decreased 5-year survival rates [19].

**Figure 3:**
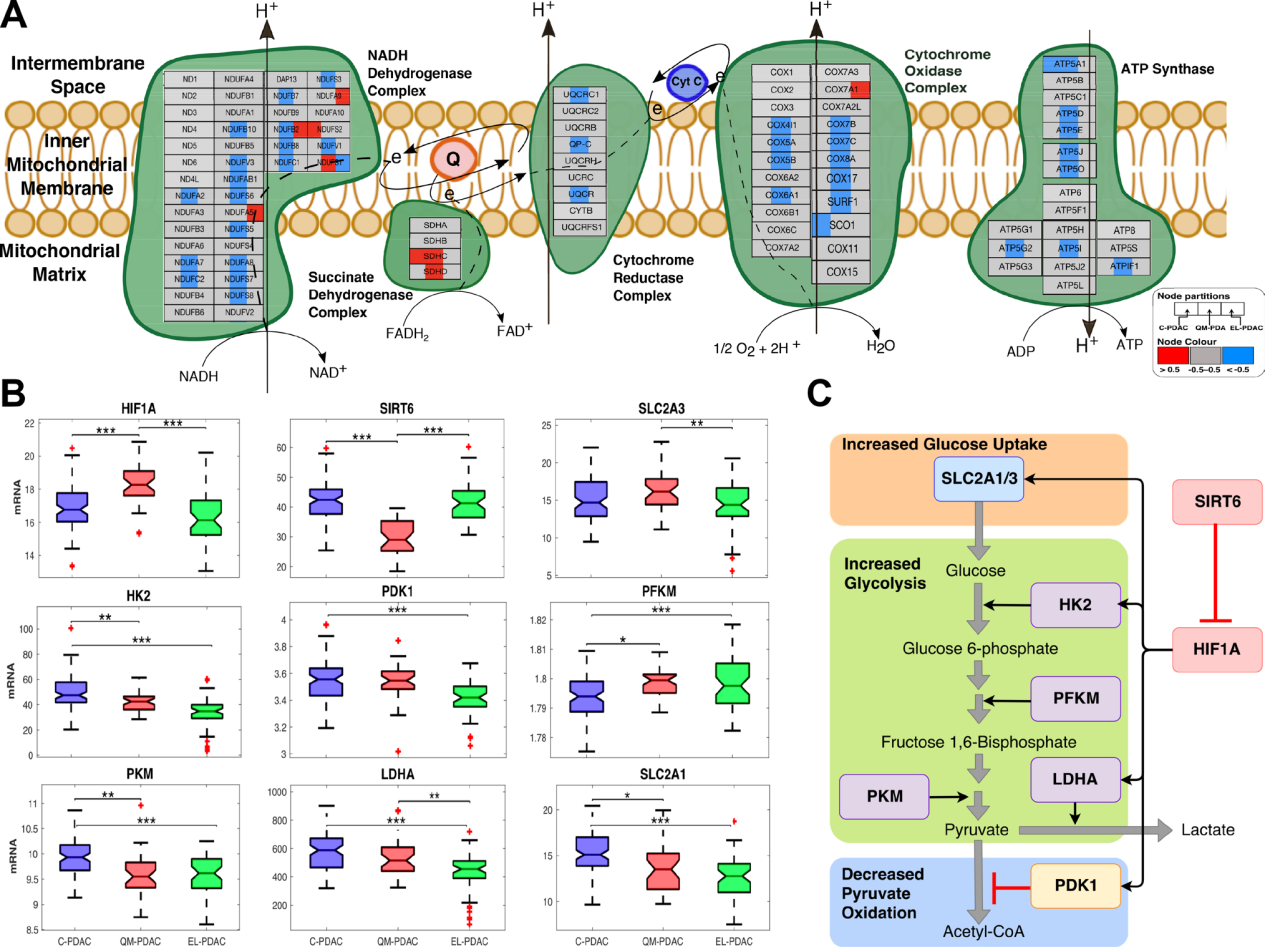
(**A**) PDAC subtype-specific electron transport chain activity: a comparison of electron transport chain activity between pancreatic cancer subtype based on mRNA expression data. Node denote genes— left section = C-PDAC, middle section = QM-PDA and right section = EL-PDAC tumours. Node are coloured based on overall subtype mRNA-expression z-score (blue = low, grey = no change, and red = high). Edges represent various types of protein interaction (refer to legend to full notations for all edges). (**B**) PDAC subtype-specific transcript levels of Warburg effect associated mRNA: levels were compared between the tumour subtypes using one-way analysis of variance. The data are where transformed using the Box-Cox transformation. ^***^, ^**^, and ^*^, denote pairwise student *t*-test statistical significance for *p* values of 0.001, 0.01 and 0.05, respectively. On each box, the central mark indicates the median, and the bottom and top edges of the box indicate the 25th and 75th percentiles, respectively. The whiskers extend to the most extreme data points not considered outliers, and the outliers are plotted individually using the '+' symbol. (**C**) Control of glucose metabolism by HIF1A: SLC2A1, SCL2A3, HK2, LDHA, PDK1 are positively regulated (black arrows) by HIF1A. PDK1 negatively regulates (red blunt arrows) the conversion of pyruvate to acetyl-CoA.

Further, we observed reduced expression of genes involved in electron transport chain and oxidative phosphorylation in the QM-PDAC tumours and, to a lesser degree, in the C-PDAC tumours (Figure 2A and Figure 3A). Since patients with QM-PDAC and C-PDAC tumours exhibited the worst clinical outcomes, these findings are consistent with the hypothesised link between the Warburg effect (characterized by decreased mitochondrial respiration and increased glycolytic activity) and tumour aggressiveness [20, 21]. It is well established that hypoxia-inducible factor 1 (HIF-1), which regulates glucose homeostasis by controlling the expression of multiple glycolytic genes and glucose transporters [22, 23], drives the Warburg phenotypes of various cancers, including those of the lungs and clear renal cells [22, 24].

In support of our findings that QM-PDAC tumours have a Warburg phenotype, we found elevated levels of *HIF1A* and concomitantly lower levels of its corepressor, *SIRT6* (Figure 3B) [25]. Also, we found higher transcript levels of *SLC2A1* and *HK2* in C-PDAC tumours relative to EL-PDAC tumours and the highest *SLC2A3* transcript levels in QM-PDAC tumours. The transcription of *SLC2A1*, *SLC2A3* and *HK2* is up-regulated by HIF1A [25]. Whereas *SLC2A1* and *SLC2A3* respectively encode the glucose transporters, GLUT1 and GLUT3, *HK2* encodes hexokinase II. Interestingly, among the class 1 GLUT transporters GLUT3 has the highest affinity for glucose and among the hexokinase isoforms, hexokinase II has the highest catalytic efficiency. Further, GLUT 3 and hexokinase II are both reportedly elevated in various cancers [26–28]. This is particularly significant as the combined action of GLUT3 and hexokinase II should afford tumour cells preferential access to available glucose for energy production via glycolysis.

We also found that the transcript levels of certain key glycolytic pathway enzymes varied between PDAC subtypes: these included the transcript levels of pyruvate kinase (*PKM*), lactate dehydrogenase (*LDHA*) and pyruvate dehydrogenase complex kinase-1 (*PDK1*) which were highest in C-PDAC tumours and lowest in EL-PDAC tumours (Figure 3B). Recently, studies have shown that the HIF-1A induced expression of PDK1 limits the oxidation of pyruvate to acetyl-CoA by inhibiting the pyruvate dehydrogenase complex in cancers of the breast and kidney [29, 30]. Accordingly, we suggest that in QM-PDAC and C-PDAC tumours, the upregulation of *PDK1* and *LDHA* would likely favour the conversion of glucose-derived pyruvate to lactate, thereby promoting the Warburg effect in these PDAC subtypes (Figure 3C).

To further investigate the degree of EGFR and TGF-β signalling pathway activation in the different PDAC subtypes, we mapped mRNA expression levels onto these pathways. We observed that there were higher mRNA levels for genes involved in these pathways in the QM-PDAC tumours than in the C- and EL-PDAC tumours (Supplementary Figures 3 and 4). Here and subsequently, we opted to compare QM-PDAC to C-PDAC and EL-PDAC tumours (referred to collectively as the ”other” subtypes) because QM-PDAC has the most distinct molecular signature of the three PDAC subtypes. Many kinases have been previously implicated in carcinogenesis and, accordingly, we identified variations between the PDAC subtypes in the mRNA levels of the various kinases that are involved in the EGFR and TGF-β pathways [31]. We used an unbiased computational approach called Expression2Kinases (X2K) to identify the kinases that might be driving the hyperactivation of the EGFR and TGF-β pathways in QM-PDAC tumours. Among the top ten kinases identified were six well-documented oncogenes (*AKT1, GSKB, MTOR, MAPK1, MAPK14*, and *MAPK7*), all of which are involved in the EGFR and TGF-β pathways (Figure 2B and 2C) [32]. Also present in the top ten list were CDK1, CDK2 and ATM: kinases which are involved in cell cycle control.

### The mutational landscape of PDAC subtypes

**Figure 4:**
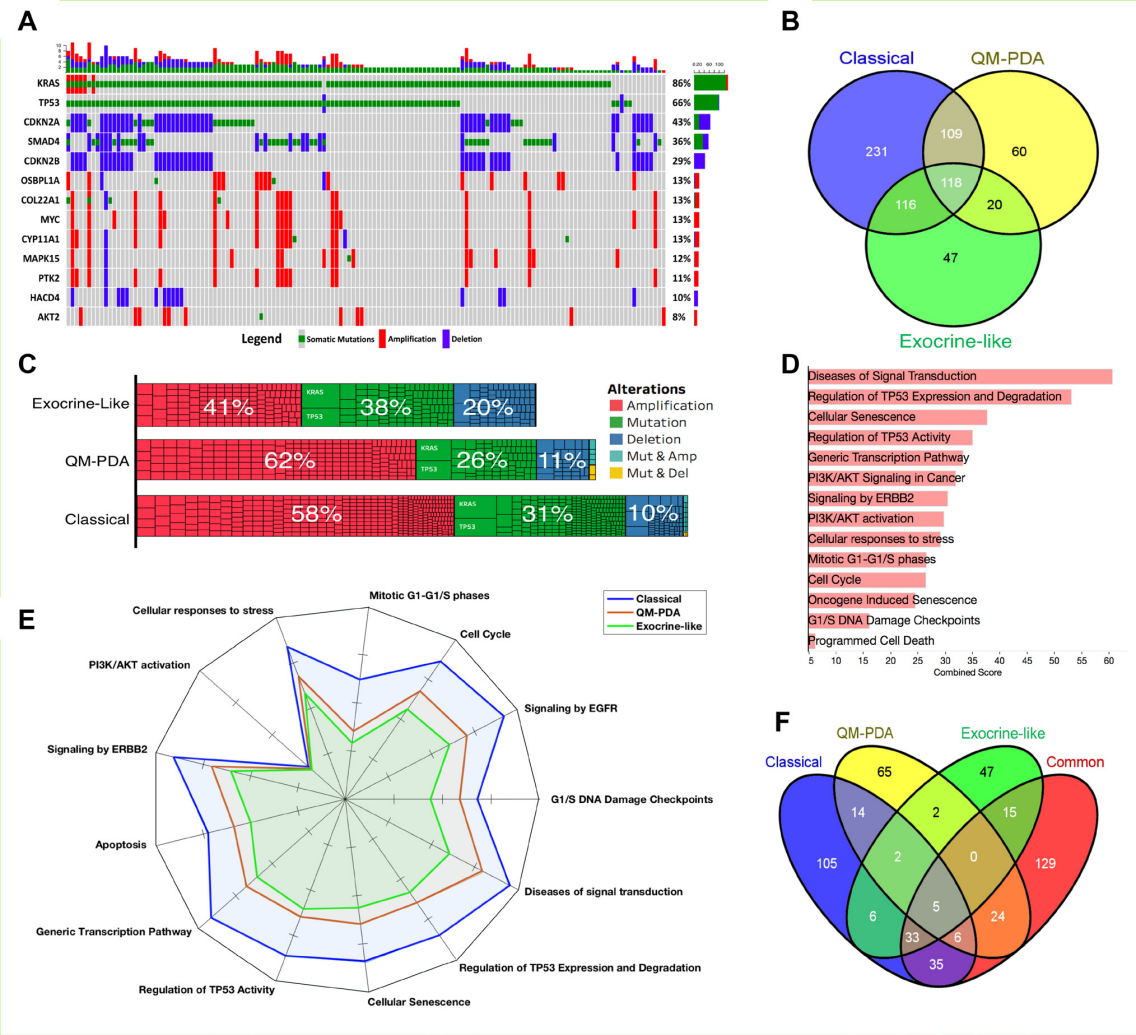
Mutation and gene copy number analyses (**A**) Genes with the most alterations in PDAC tumours. The only genetic alterations considered are mutations in, and amplifications of known oncogenes, and mutations in, and deletions of, known tumour suppressor genes. (**B**) The distribution of alterations among the three PDAC subtypes. Refer to Supplementary File 5 for details concerning alterations in each set. (**C**) The extent of genomic alterations expressed as a percentage of total numbers of alterations found within the tumours of each PDAC subtype. Each cell in the bar-grid represents a mutant gene. (**D**) Reactome pathway enrichment results of the 118 genes that are commonly altered in tumour cells of all three PDAC subtypes. Refer to Supplementary File 6 for the complete list of Reactome pathways that represent significantly enriched genetic alterations in the different PDAC subtypes. (**E**) The predicted extent of mutation-induced pathway dysregulation for the different PDAC subtypes. (**F**) The distribution of mutation-induced pathway dysregulations for mutations specifically associated with particular PDAC subtypes and common pathway enrichment. The non-overlapping pathways are those disrupted only in single PDAC subtypes (see Supplementary File 6).

We evaluated the scope of genomic alterations in PDAC subtypes by focusing on the types of genetic changes that are known to promote oncogenesis. Specifically, these encompassed gain-of-function mutations in oncogenes (OGs), amplification of OGs, loss-of-function mutations in tumour suppressor genes (TSGs), and deletions in TSGs. Across all the PDAC subtypes we found that, as has been reported elsewhere, *KRAS*, *TP53*, *CDKN2A*, *SMAD4*, and *CDKN2B* were the most commonly altered genes (Figure 4A) [1, 2, 8].

SMAD4 signals through the canonical TGF-β pathway and therefore deletions in *SMAD4* should limit signalling through this pathway [33]: a factor that may seem inconsistent with our earlier finding that genes in this pathway display elevated levels of transcription (Figure 2C and Supplementary Figure 4). However, SMAD4 loss does not initiate tumorigenesis in human pancreatic cancers [33–35]. Further, in pancreatic tumours displaying either *SMAD4* deletions or SMAD4 under-expression, ligand stimulation of the TGF-β pathways activates non-canonical pathways including the MAPK, p38/JNK, and PI3K-mTOR pathways which can function independently of SMAD signalling [33, 36, 37]. Moreover, we found that among the PDAC subtypes, QM-PDAC had the lowest transcript levels of genes in the SMAD gene family (SMAD2/3/4/7; Supplementary Figure 4). Therefore, consistent with other studies, these results indicated an association between reduced SMAD expression and poor survival [36, 38].

Whereas we observed higher gene deletion frequencies in EL-PDAC tumours than in QM-PDAC and C-PDAC tumours, QM-PDAC and C-PDAC tumours displayed higher gene amplification frequencies (Figure 4C). Across all the PDAC subtypes, 118 genomic alterations were observed, mostly impacting genes involved in diverse cell signalling pathways (Figure 4B and 4D); a finding consistent with the hypothesis that, like most other cancers, PDAC is primarily a consequence of disrupted signal transduction pathways (Figure 4D, 4E) [11, 39].

We uncovered little overlap in the signaling pathways that were impacted by genetic alterations that were observed in only one of the PDAC subtypes (Figure 4E and 4F, also see Supplementary Figure 6). We found that 62% of the pathways affected by mutations were altered in tumours belonging to at least two PDAC subtypes, whereas only 18%, 11%, and 8% of altered pathways were unique to C-PDAC, QM-PDAC, and E-PDAC tumours, respectively. This suggests that only a small proportion of mutations contribute to differences in the signaling pathway perturbations that are seen between the subtypes. Conversely, it therefore also suggests that most of the oncogenesis promoting mutations perturb pathways that are active in all three of the PDAC subtypes.

### Integrative pathway analysis

**Figure 5:**
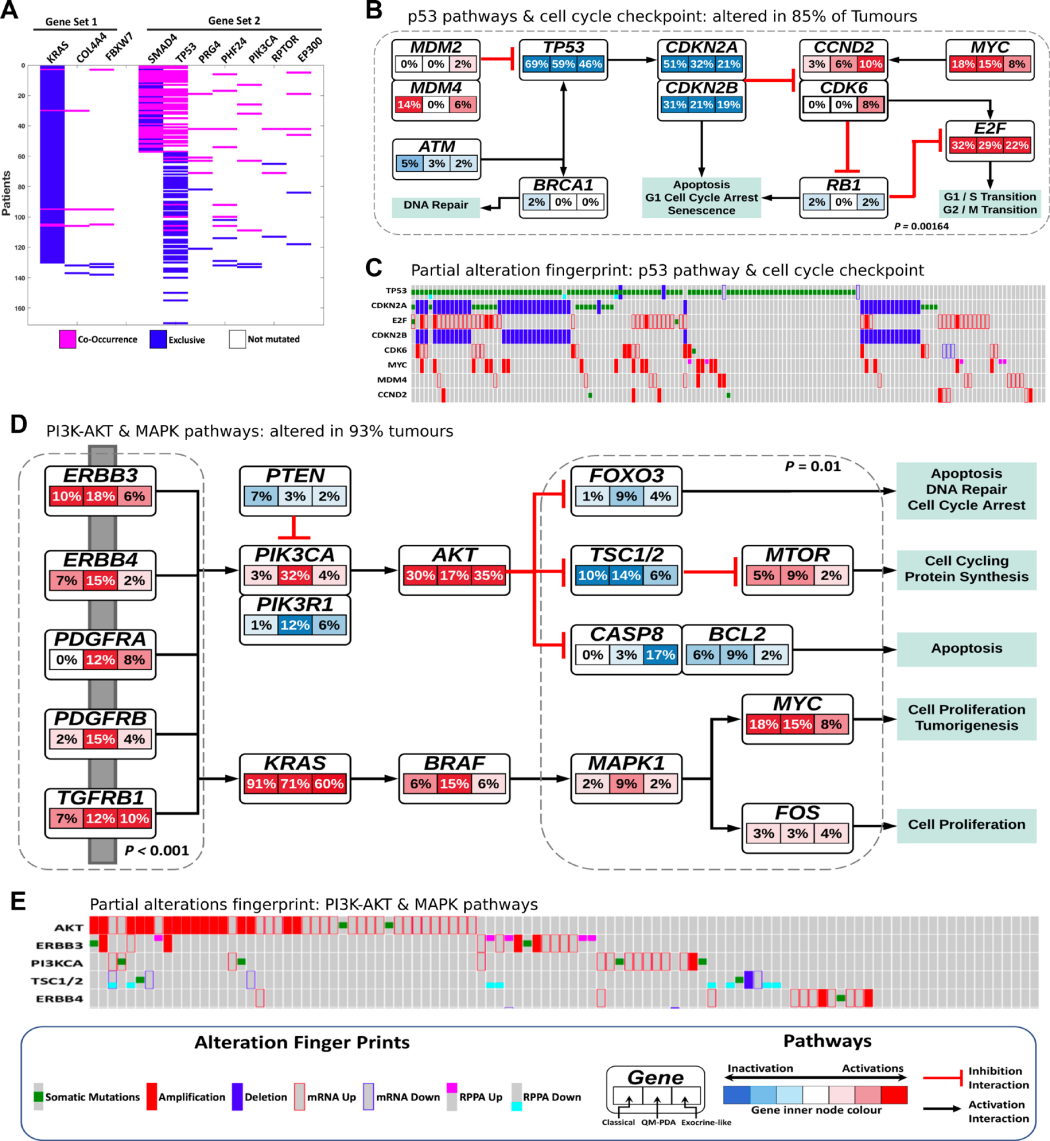
(**A**) The two co-occurring pathways in PDAC that are predicted to drive oncogenesis based on the mutational landscape representing two common driver pathways. (**B**) Alterations in p53 and cell cycle checkpoint pathways. *p*-value = C-PDAC vs Other subtypes, calculated using Fisher's exact test. Red indicates activating genetic alterations whereas blue indicates inactivating alterations. Darker shades correspond to higher alteration frequencies. Each node within the pathway represent a gene and the highlighted segments within each node and the percentage representing the alteration in the three PDAC subtype: C-PDAC, QM-PDAC and EL-PDAC from left, centre, and right, respectively. (**C**) The pattern of genetic alteration in selected genes that encode proteins involved in the p53 and cell cycle checkpoint pathways. (**D**) Alterations in the MAPK, RTKs and PI3K signaling pathways. *p*-values = QM-PDAC vs Other subtypes, calculated using Fisher's exact test. The connectivity of network components was extracted from Reactome Pathways, BioGrid and the literature. (**E**) The pattern of genetic alterations in selected genes that encode components of the MAPK and PI3K-mTOR pathways.

We used the co-occurring mutated driver pathway (CoMDP) mathematical algorithm to discover *de novo,* two co-occurring pathways that may be driving the progression of PDAC; the first pathway involved the genes *KRAS, COL4L4 and FBWX7*, and the second involved the genes *SMAD4, TP53, PHF24, PRG4, PI3KCA, RPTOR and EP300* (Figure 5A) [40]. We expanded the CoMDP solution pathways using known protein-protein interactions to generate a network enriched with *MAPK, PI3K, and TP53* pathway members (Supplementary Figure 7). Two of the genes in the CoMDP solution pathways, *PRG4* and *PHF24*, did not map to any signal transduction pathway, emphasising the fact that the roles of some potentially key proteins in oncogenesis still need de be defined. In particular, virtually nothing is known about *PHF24* [41].

To further investigate the degrees of alteration that are evident in the expanded CoMDP solution networks for the different PDAC subtypes, we mapped a combined dataset of mRNA transcript levels, protein expression levels, mutations and CNA onto these networks. We found that alterations in p53 and cell cycle checkpoint pathway genes were most apparent in C-PDAC tumours (Figure 5B and 5C), whereas alterations in specific MAPK and PI3K-mTOR pathway genes were more apparent in QM-PDAC tumours (Figure 5D and 5E). We found that the PI3K-mTOR and MAPK pathways were altered in 93% of all PDAC tumours. Alterations, included the activation of, among others, the PI3KCA (in 13% of tumours), AKT (in 27%) and BRAF (in 9%) genes, and the inactivation of the PTEN (in 4% of tumours), TSC1/2 (in 7%) and FOXO3 (in 5%) genes. Alterations in the *PI3KCA* oncogene and its negative regulator, *PTEN,* occur in cancers of the colon, breast, and prostate: cancers where the co-occurrence of *PI3KCA* and *PTEN* mutations appears to both drive oncogenesis, and reduce anticancer drug sensitivity [10, 42]. Furthermore, we observed that PIK3R1 (the regulatory subunit of PI3K) was inactivated in 6% of PDAC tumours; inactivated PIK3R1 promotes the phosphorylation of AKT, which itself promotes oncogenesis as it activates numerous OGs and inhibits TSGs within the cell [43, 44].

Alterations in the p53 and cell cycle pathways are frequent in cancer, and here we found that such changes were apparent in 85% of the tumours examined [45]. In addition to activated MDM2 and MDM4 (which both inhibit p53 activity), MYC, and CDK2/4/6, we found that p53 was inactivated in 58% of all tumours. Similarly, CDKN2A, CDKN2B and ATM (a kinase that activates p53) were inactivated in 35%, 42% and 3% of all tumours, respectively [45, 46]. This suggests that, within the p53 and cell cycle checkpoint pathways, hyperactivated OGs such as MYC, MDM2, CDKs and inactivated TSGs such as TP53, CDKN2A and CDKN2B, may act together to promote oncogenesis both by limiting the repair of damaged DNA, and by permitting affected cells to proliferate uncontrollably through the inhibition of apoptosis [45, 46].

Consistent with previous observations, we found mutations, CNAs and changes in mRNA transcription and protein expression levels for proteins that participate in various signalling pathways that have previously been associated with pancreatic cancer [2, 8, 10]. Specifically, we observed alterations in the Notch (61%), apoptosis (32%) and NF-kβ (19%) pathways (Supplementary Figure 8). Also, we found a variety of alterations in 41 genes that encode the ATP-binding cassette (ABC) transporter proteins in 72%, 82% and 79% of all tumours that belong to the C-PDAC, QM-PDAC and EL-PDAC subtypes, respectively (Supplementary Figure 8D). The most altered ABC transporter gene in any PDAC subtype was *ABCC9*; found altered in 21% of all in QM-PDAC tumours. Overall, we observed that 78% of all PDAC tumours harboured a genetic alteration in at least one ABC transporter gene. ABC transporter-mediated energy-dependent efflux of a multitude of unrelated classes of anticancer drugs across membranes is a major cause of multidrug resistance and chemotherapeutic failures during cancer therapy [47, 48]. Therefore, future efforts to determine tumour cell ABC transporter gene mutations that accentuate the activities of their encoded transporters are expected to guide precision medicine [48].

### Connectivity of genomic alterations to transcription factors and their pathway activities in pancreatic cancer

To link genomic changes to transcriptional events, we applied the Tied Diffusion Through Interacting Event (TieDIE) approach to reveal a protein interaction subnetwork that connects altered genes to transcription factors and their putative targets [49]; a network referred to below as the TieDIE subnetwork. Additionally, we used the PARADIGM-shift algorithm to infer pathway activity levels of all proteins that are known to participate in various signaling pathways in each of the three PDAC subtypes [50]. Furthermore, using heat diffusion analysis from the MAPK1 and TP53 nodes of the TieDIE subnetwork, we extracted two pathways that recapitulated signaling via the MAPK and p53 pathways.

**Figure 6:**
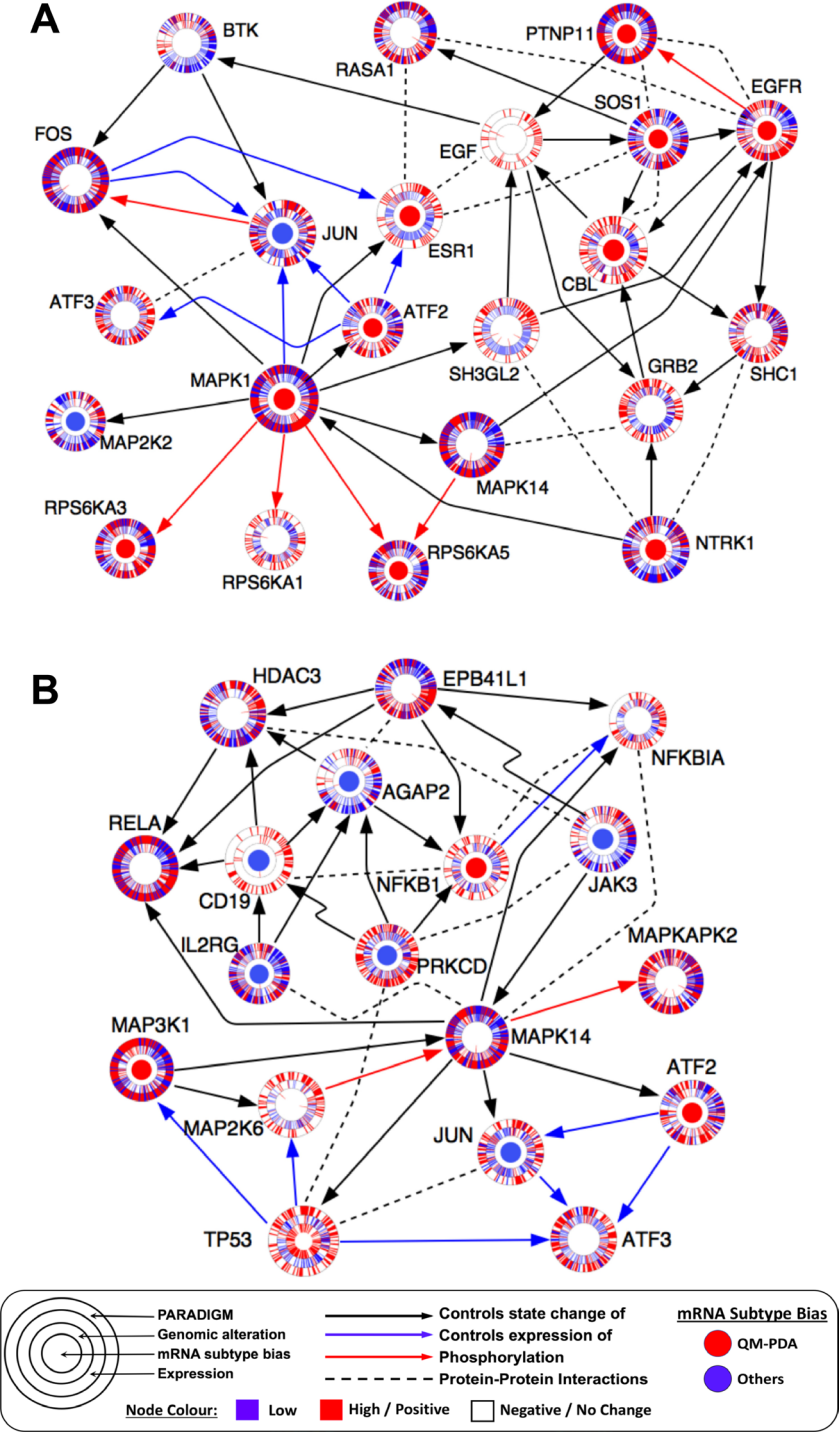
(**A**) MAPK heat diffusion sub-network: pathway extracted from the TieDIE subnetwork using heat diffusion analysis from the MAPK1 network node. (**B**) p53 heat diffusion sub-network: pathway extracted from the TieDIE solution network using heat diffusion analysis from the TP53 network node. Each node indicates a pathway protein shown as concentric rings. The inner node denotes differential mRNA expression (Benjamini-Hochberg adjusted *p* < 0.05) bias for genes when comparing the QM-PDAC subtype tumours to those of the other PDAC subtypes (red = QM-PDAC bias, blue = bias towards other subtypes). The second ring indicates the presence of genomic alterations for that gene in each patient’s tumour, with each patient’s tumour being denoted by a spoke within the ring. The third ring shows mRNA expression levels for each tumour sample (red = high, blue = low). The outer ring indicates the PARADIGM inferred pathway activity for that protein in each tumour sample (red = high, blue = low). Arrows indicate known protein-protein interactions extracted from UCSC Super pathway, KEA, ChEA or inferred from the literature. We have attempted to make the visualisation clearer by omitting some interactions between some network nodes.

The MAPK1 network was enriched with proteins whose associated mRNA transcription levels were significantly higher in QM-PDAC tumours compared to other PDAC subtypes. These proteins included EGFR (a receptor of the EGFR pathway that we found activated in QM-PDAC tumours), SOS1 and GRB2 (both of which are upstream signalling proteins in the canonical MAPK signalling pathway; Figure 6A) [51]. Also, the MAPK network connected TFs that are induced upon activation of the MAPK pathway, to proteins which are known to promote oncogenesis (such as FOS, JUN, ATF2 and ESR1). This inferred connectivity was further supported by the PARADIGM analysis which predicted that ESR1, JUN, GRB2 and CBL would have high degrees of activity [51, 52].

The p53 network, on the other hand, was more prominent in C-PDAC and EL-PDAC tumours, and it connected signalling proteins to various TFs that are known to promote carcinogenesis, including ATF3, PRKCD, NFKB1 and NFKBIA [53–55].These TFs were also predicted by PARADIGM to have a high degree of activity (Figure 6B).

Collectively these analyses emphasise that certain pathways may be more prominent than others in the different PDAC subtypes. Differences between the PDAC subtypes in the activity of specific signal transduction proteins suggests that some of these proteins could be targets of PDAC subtype-specific anti-cancer drugs (see Supplementary Figure 10 and Supplementary Table 2).

## DISCUSSION

Through comprehensive transcriptomic and integrative profiling of pancreatic cancer, we have uncovered various functional alterations and signaling pathway perturbations and revealed how these alterations and perturbations might be associated with clinically relevant differences between patients with different PDAC subtypes. In particular, the discovery that QM-PDAC tumours are characterised by what is likely to be ESR1 and NTRK1 transcription factor-mediated over-activation of genes associated with the EGFR and TGF-β pathways (Figure 6A), provides a rationale to target these tumours with drugs that either downregulate ESR1 and NTRK1, or inhibit EGFR and TGFBR2 (Supplementary Figure 10) [56, 57].

Furthermore, we find that, in general, PDAC is characterised by pervasive RTK, MAPK and PI3K-mTOR alterations [1, 8, 58, 59] and that over 90% of the potentially oncogenic alterations occur in genes that are directly involved in the RTK, MAPK and PI3K-mTOR signalling pathways. It is well established that PDAC tumours frequently respond to MAPK and/or PI3K-mTOR pathway inhibitors [58, 60]. While cases where these inhibitors have failed to provide therapeutic benefits have highlighted the heterogeneity of PDAC, they have also emphasised the importance of finding additional drugs that are either more generally applicable to PDAC treatment, or which can be used to target the signalling pathways that are most relevant for specific PDAC subtypes [60–62]. We identified that the most prominent cell signalling changes in EL-PDAC and C-PDAC tumours were within the p53 and cell cycle checkpoint pathways; hinting that these tumours might respond to cell cycle inhibitors. Consistent with our findings, other PDAC studies have also reported co-occurring mutations in genes involved in the p53 and cell cycle pathways. Collectively these studies provide a rationale for potentially treating PDAC using an approach that synchronously targets all of these pathways [63–65]. Furthermore, we have uncovered other receptors, intermediary signal transduction proteins and TF targets that may drive oncogenesis: all of which could be targeted by drugs designed to specifically treat QM- C- or EL-PDAC tumours.

Alterations in metabolism and cellular bioenergetics are hallmarks of cancer cells and represent an active area of research that is anticipated to yield novel anti-cancer drugs that could be used in combination with targeted-therapies or chemotherapy [66–68]. Here, we found that QM-PDAC and, albeit to a lesser extent, C-PDAC tumours exhibit a Warburg metabolic phenotype (Figure 3) [64]. Associations between the Warburg phenotype and both increased disease aggressiveness and poorer clinical outcomes have been previously reported [64, 67]. As expected, we observed decreased overall survival and a shorter duration of disease-free survival in patients with QM- and C-PDAC tumours (i.e. tumours with the Warburg phenotype) relative to patients with EL-PDAC tumours (i.e. those without the Warburg phenotype). In this regard, scrutinising the metabolic differences between PDAC tumour subtypes is likely to yield further leads for the development of novel therapeutic approaches.

We observed that most of the genomic alterations which are found within PDAC tumour cells are found in tumours belonging to all three of the defined PDAC subtypes. This finding suggests that improved responses to targeted-therapies may be achievable by systematic targeting of hub kinases within the multiple alternative signalling pathways that enable cancer cells to frequently acquire resistance [69, 70].

By integrative analyses of genomic, transcriptomic, and proteomic data, we have uncovered novel signalling pathway aberrations that exist in PDAC tumour cells at the DNA, mRNA and protein levels. Although the mutational landscape of a particular tumour could influence drug efficacy, recent studies of a range of other cancers employing approaches similar to those used here have identified an array of altered pathways, each of which could present either new cancer drug targets or clinically relevant biomarkers of disease [9, 71]. Altogether, our analyses have revealed widespread signaling network perturbations in PDAC subtypes, many of which could likely impact treatment outcomes and which are therefore also potential targets for novel anticancer drugs.

## MATERIALS AND METHODS

We obtained data for 185 PDAC patients involved in the TCGA project. Besides treatment outcomes these data include: whole exome sequence (WES; *n* = 185), transcriptome data (determined using RNAseq; *n* = 179); DNA copy number and mutation data (*n* = 179), and targeted proteome data (determined using RPPA; *n* = 123). Not all types of data were available for all patients because of assay failures, incomplete specimen availability and quality of issues with certain samples. All data used in our analyses are available from the TCGA website; https://portal.gdc.cancer.gov/repository.

### Transcriptome-based classification

We performed unsupervised hierarchical clustering on RNA-seq data to identify three distinct PDAC subtypes. Before clustering, we removed data for unexpressed genes and genes that exhibited little variation between patients. To return only exemplars for each cluster, we applied an anomaly detection algorithm based on an approximate Gaussian distribution [72]. Finally, we further validated the consistency of tumours within each cluster using a support vector machine classifier which yielded an average 10-fold cross validation classification accuracy of 95.5% over ten models (Supplementary Figure 1A). Using the transcriptomic classification framework established by Collision *et al.* [15], we classified the pancreatic cancer clusters as C-PDAC, QM-PDAC and EL-PDAC; respectively corresponding to clusters 1, 2 and 3 [15]. We have summarised the distribution of tumour grades across these PDAC subtypes in Supplementary Figure 1B.

### Treatment outcomes

We integrated mRNA expression-based classification of PDAC subtypes with clinical information to review tumour characteristics specific to each of the PDAC subtypes. The Kaplan–Meier method was used to estimate overall survival and the duration of disease-free survival in a pairwise manner between subtypes [73]. Furthermore, the Fisher exact test was used evaluate associations between tumour subtypes and various clinical variables including treatment outcomes at the first, and later courses of treatment.

### Differential gene expression, functional and pathways analyses

The identification of differentially expressed genes was performed in MATLAB using an implementation based on the negative binomial model (see Supplementary File 1) [74, 75]. Gene set enrichment analysis (GSEA) was employed to extract knowledge of overrepresented Gene Ontology (GO) terms for various functional processes and signalling pathways between molecular subtypes [16]. Complete GSEA results are provided in Supplementary Files 2A–2C, for C-PDAC vs QM-PDAC, C-PDAC vs EL-PDAC and QM-PDAC vs EL-PDAC, respectively. Visualisation of significantly enriched GO terms of functional process and signalling pathways between subtypes was done in the Cytoscape plugin, Enrichment Map [76]. Furthermore, the mapping of gene expression levels onto template WikiPathways of the EGFR and TGF-β signalling pathways and the electron transport chain was done using the software PathVisio 3 (See Figure 3A, Supplementary Figure 3 and 4) [77]. For this, we used z-score normalised expression data categorised into three levels: 1) Low for z-scores below −0.5; 2) no change for z-scores between −0.5 to 0.5; and 3) high for z-score above 0.5. The highlighted scale was chosen to consistently capture variations in gene expression across entire pathways.

### Prediction of regulator kinases

We computationally predicted upstream regulatory kinases that likely effect the observed differences in the gene expression signatures between QM-PDAC and the other PDAC subtypes using Expression2Kinases (X2K) [78]. X2K employs a reverse engineering network-based approach to predict upstream regulatory kinases based on prior knowledge. We obtained a list of differentially expressed genes between QM-PDAC and the others PDAC subtypes: 242 up-regulated genes and 1011 down-regulated genes based empirical Bayes statistics. Using this gene list, we predicted upstream regulatory TFs that are likely to be responsible for the observed changes in gene expression using the Chromatin Immunoprecipitation (ChIP) Enrichment Analysis (ChEA; 2016) [79]. In the next analysis, we linked the top 10 predicted TFs to upstream regulatory mechanisms by generating a TF-intermediate protein-protein interaction sub-network based on prior knowledge (Supplementary Figure 5A). Finally, we analysed the sub-network for enriched targets of known protein kinases that are likely to phosphorylate proteins within the sub-network using the Kinase Enrichment Analysis (KEA; 2015) [80]. See supplementary file 3 for a full list of computationally predicted kinases and their rankings.

### Mutation and copy number alteration analyses

We evaluated the scope of genomic alterations in PDAC subtypes using significantly mutated genes and copy number alteration identifications obtained from MutSigCV and GISTIC2.0 outputs, respectively [81, 82]. Data for the genomic alteration analysis was processed as follows: oncogenes (OGs) and tumour suppressor genes (TSGs) in the samples were annotated using information from multiple sources. These include the Sanger Consensus Cancer Gene Database (699 OGs and TSGs), the UniProt Knowledgebase (304 OGs and 741 TSGs), the TSGene database (1,219 TSGs) and the ONGene database (725 OGs) [34, 86–88] [32, 83–85]. Collation of data from these sources yielded a list of 3,688 OGs and TSGs, representing 2,773 unique genes (969 OGs and 1,804 TSGs). We utilised this list of OGs and TSGs to extract genetic changes anticipated to have a potential impact on the oncogenesis of pancreatic cancer. Explicitly, we returned only gain-of-function mutations and gene amplifications for known OGs. Also, for known TSGs, we returned loss-of-function genetic changes that involve mutations and deletions. Using this processed data, we identified frequently altered genes that likely have detrimental impacts concerning pancreatic carcinogenesis (Figure 4). We compared gene mutations between the PDAC subtypes to generate lists of mutations that are common among subtypes or unique to particular subtypes (see Supplementary File 4). Using these lists, we performed a Reactome pathway enrichment analysis by querying Enrichr either with genes that were consistently altered in tumours of all three PDAC subtypes, or with genes that were altered in only one of the PDAC subtypes (see Supplementary File 5 and Supplementary Figure 6A–6C) [86].

### Integrative analysis of expression and genomic alterations

#### Identification of Co-occurring driver pathways

To discover driver pathways based on the patterns of mutations associated with PDAC, we applied the CoMDP algorithm which employs a mathematical programming method to identify *de novo* driver pathways in cancer from mutation profiles [40]. Briefly, this method identifies pathways that have a set of mutated genes with both high coverage (i.e. present in the tumours of multiple individuals) and high exclusivity, and the pathways exhibit a statistically significant co-occurrence pattern. Using mutation data, we ran the CoMDP test with *K* = 5 to 11 (*K* equals the total gene set size) to return mutated driver pathways for all *K* values (Supplementary Table 1). Genes in the CoMDP solution for *K* = 10 were connected using experimentally verified protein-protein interactions to generate an intermediary network (Supplementary Figure 7). The solution network was enriched with members of the PI3K, MAPK, p53 and cell cycle regulation pathways. To visualise the extent of pathway aberration at DNA, mRNA and protein levels, we mapped genomic alteration data, mRNA transcript abundance data and protein expression data onto the network. For the genomic alteration data, we only considered gain-of-function mutations and gene amplifications for the OGs, and loss-of-function mutations and deletions for the TSGs. For the transcription and protein expression data we only considered OGs that had a degree of upregulation indicated by a >2 Z-score and for the TSGs a degree of downregulation indicated by a <−2 X-score. The generated combined dataset was mapped on signaling pathways over-represented in the CoMDP solution network expanded using a prior-knowledge network. Additionally, plots of alteration patterns in genes among tumours were generated using the R package complex heatmaps [40, 87]. Mapping of alterations onto genes in pathways shown in Supplementary Figure 8A–8C were done using the software PathwayMapper [88].

### Inferring gene activity from pathway analysis of copy number and expression data

We used PARADIGM-shift, a probabilistic graphical model approach that infers the activity of signalling pathway proteins by detecting differences in the expected activity of a protein on its downstream target relative to what is expected given it upstream modulator [50]. We ran PARADIGM with default settings using three datasets as inputs: (i) a dataset including only statistically significant CNA as determined by GISTIC2, (ii) a normalised gene expression dataset matching the CNA input file, and (iii) a custom UCSC Pathway formatted file. Pathway information of known gene interactions was created from various sources including Reactome pathways, KEGG Pathways, the KEA database, the ChEA database and the UCSC Super pathway [79, 80, 89, 90]. PARADIGM predicted integrated pathway levels results are provided in Supplementary File 6.

### Identification of genomic perturbation associated with transcriptional changes

Genomic perturbations in PDAC subtypes were connected to associated transcriptional changes using TieDIE [49]. This method uses a heat diffusion process to identify relevant pathways that might be altered in tumours. To reveal sub-networks that distinguish QM-PDAC from the other PDAC subtypes, using genes that we found altered in at least 5% of all tumours, we generated a ranked list of genes that were differentially mutated between QM-PDAC tumours and those of the other PDAC subtypes using the Fisher’s exact test. The resulting genes are assumed to be responsible for the distinctive molecular signatures between subtypes—these were used as upstream inputs in TieDIE. A downstream input file was generated by computationally identifying the TFs that are most likely to be responsible for the difference in the transcriptome signatures between the QM-PDAC tumours and those of the other PDAC subtypes. The upstream and downstream input files, together with a custom super pathway, were used in TieDIE to compute a subnetwork connecting genomic alterations to transcriptional events (Supplementary Figure 9). We performed a secondary heat diffusion query on the TieDIE solution sub-network from the MAPK1 and TP53 nodes. Both MAPK1 And TP53 were flagged as being of likely importance based on both the numerous alterations of these genes within PDAC tumours and the pathway analysis that we had previously performed. These analyses produced two subnetworks that recapitulate signaling through the MAPK1 and TP53 pathways to their downstream TFs.

### Statistical analyses

Except were stated otherwise all statistical analyses were performed in MATLAB 2017b. The Fisher’s exact test was used assess associations between categorical variables. The Wilcoxon rank sum test and Kruskal–Wallis test or independent sample Student *t*-test and One-way ANOVA were used for continuous variables were appropriate. Statistical tests were considered significant at *p* < 0.05 for single comparisons, and Benjamini-Hochberg adjusted *p*-values for multiple comparisons.

## SUPPLEMENTARY MATERIALS FIGURES AND TABLES



















## Abbreviations

TCGA: The Cancer Genome Atlas
PDAC: Pancreatic Ductal Adenocarcinoma
QM-PDAC: quasi-mesenchymal PDAC
EL-PDAC: exocrine-like PDAC
C-PDAC: classical PDAC
mRNA: Messenger Ribose Nucleic Acid
RPPA: Reverse Phase Protein Array
RNA-Seq: RNA-sequencing
CNA: Copy Number Alteration
EGFR: Epidermal Growth Factor Receptor
GSEA: Gene Set Enrichment Analysis
TGF-β: Transforming Growth Factor–Beta
PI3K: Phosphoinositide 3-Kinase
mTOR: Mammalian Target of Rapamycin
MAPK: Mitogen Activated Protein Kinase
TFs: Transcription Factors
OGs: Oncogenes
TSGs: Tumour Suppressor Genes
