# Supplementary material for: Integrative landscape of dysregulated signaling pathways of clinically distinct pancreatic cancer subtypes
Source: Oncotarget. 2018 Jun 26;9(49):29123–39. doi: 10.18632/oncotarget.25632 (PMC6044387; doi:10.18632/oncotarget.25632)
Supplement: Supplementary file 1 [file oncotarget-09-29123-s001.pdf]

Integrative landscape of dysregulated signaling pathways of clinically distinct pancreatic cancer subtypes

SUPPLEMENTARY MATERIALS

Supplementary Table 1: Co-occurring gene sets in pancreatic cancer

K	Gene set 1	Gene set 2	n1	n2	r1, 2	Co-occurrence
**5**	*KRAS*	*SMAD4 TP53 PHF24 CDKN2A*	130	134	0.84	<0.001
**6**	*KRAS CDKN2B*	*SMAD4 TP53 PHF24 CDKN2A*	136	134	0.89	<0.001
**7**	*KRAS CDKN2B COL4A4*	*SMAD4 TP53 PHF24 CDKN2A*	138	134	0.9	<0.001
**8**	*KRAS COL4A4 FBXW7*	*SMAD4 TP53 PHF24 RPTOR EP300*	135	128	0.88	<0.001
**9**	*KRAS COL4A4 FBXW7*	*SMAD4 TP53 PHF24 RPTOR EP300 PRG4*	135	130	0.89	<0.001
**10**	*KRAS COL4A4 FBXW7*	*SMAD4 TP53 PHF24 RPTOR EP300 PRG4 PIK3CA*	135	132	0.91	<0.001
**11**	*KRAS COL4A4 FBXW7 SALL2*	*SMAD4 TP53 PHF24 RPTOR EP300 PRG4 PRDM11*	137	132	0.91	<0.001

Here K is the combined gene set size (gene set 1 and gene set 2). Gene sets 1 and 2, represent co-occurring driver pathway in pancreatic cancer. n1 and n2 denote the coverage of genomic alterations in pancreatic cancer and r1, 2 is the ratio of the common coverage to their union coverage (i.e., co-occurrence ratio). Co-occurrence P represents the *p*-value of the co-occurrence significance of both pathways.

Supplementary Table 2: Some dysregulated pathway drug targets in clinical trial

Drug	Target	Phases	Status	NCT Number
Palbociclib, Gedatolisib	CDK4/CDK6	Phase 1	Recruiting	NCT03065062
MK0752, gemcitabine, +	NOTCH	Phase 1	Completed	NCT01098344
NIS793, PDR001	TGF	Phase 1	Recruiting	NCT02947165
Nimotuzumab, Gemcitabine	EGFR	Phase 3	Recruiting	NCT02395016
MK-0646, Gemcitabine, Erlotinib	mTOR	Phase 1/2	Active, not recruiting	NCT00769483
Ixabepilone, Cetuximab	EGFR	Phase 2	Completed	NCT00383149
Irinotecan, Hydrochloride, Veliparib	ESR1	Phase 1	Recruiting	NCT00576654
Gemcitabine, Capecitabine, Erlotinib	EGFR/mTOR/PI3K	Phase 1	Completed	NCT00480584
Galunisertib, Durvalumab	TGF	Phase 1	Recruiting	NCT02734160
Everolimus, Octreotide, Acetate	mTOR	Phase 1	Completed	NCT01204476
Erlotinib, Gemcitabine, Oxaliplatin	EGFR	Phase 2	Completed	NCT01505413
Docetaxel, Irinotecan Hydrochloride	KRAS	Phase 2	Completed	NCT00042939
Cetuximab, Erlotinib, Hydrochloride	EGFR	Phase 1	Completed	NCT00397384
Cediranib, Maleate, Olaparib	ESR1	Phase 2	Recruiting	NCT02498613
Capecitabine, Cetuximab, Everolimus	mTOR/KRAS/EGFR	Phase 1/2	Completed	NCT01077986
Afatinib, Selumetinib, Docetaxel	PI3K	Phase 1/2	Recruiting	NCT02450656
Afatinib, Binimetinib, Capivasertib, +	CDK4/CDK6	Phase 2	Recruiting	NCT02465060

Targeted-therapy drug that are currently being evaluated in clinical trial for treatment of pancreatic cancer. + denotes more drug being used in combination. NCT denotes the national clinical trial. Information concerning clinical trials was obtained from www.clinicaltrials.gov.

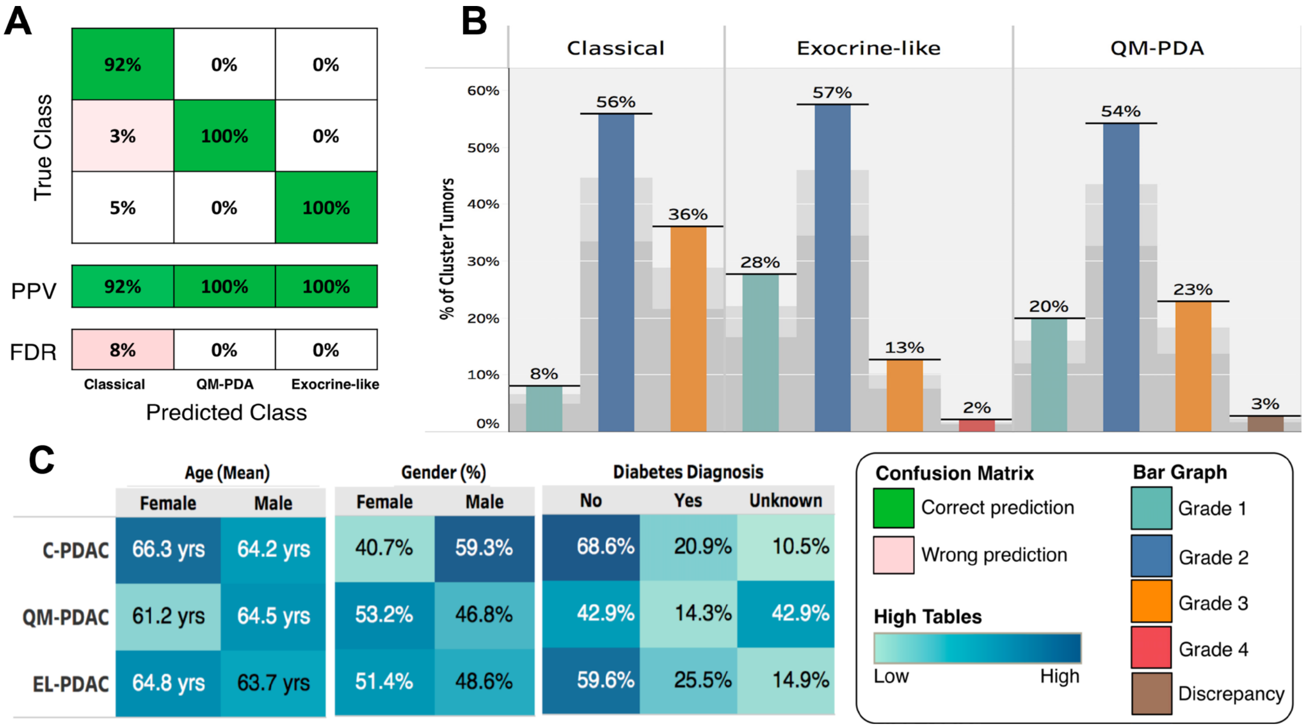

Supplementary Figure 1: (**A**) Support Vector Machine confusion matrix: a representative confusion matrix for the SVM classifier used to validated the purity of each molecular subtype of pancreatic cancer. We obtain on average a classification accuracy of 95.5% with an F1-score of 0.96. (**B**) Distribution of tumour grades across molecular subtypes: showing percentage of total count of the number of tumours for each grade broken down by molecular subtype. (**C**) Highlight tables of participant's age, gender, and diabetes diagnosis from left, centre and right respectively. The overall mean age of participants that were afflicted by C-PDAC, QM-PDAC and EL-PDAC were 65 yrs., 64 yrs. and 63 yrs, respectively: one-way ANOVA test: $F = 0.499$, $p = 0.608$. No significant association was found between gender and diabetes diagnosis, and the presence of a particular PDAC subtypes, $\chi^2 = 2.351$, $p = 0.308$, and $\chi^2 = 0.611$, $p = 0.737$, respectively.

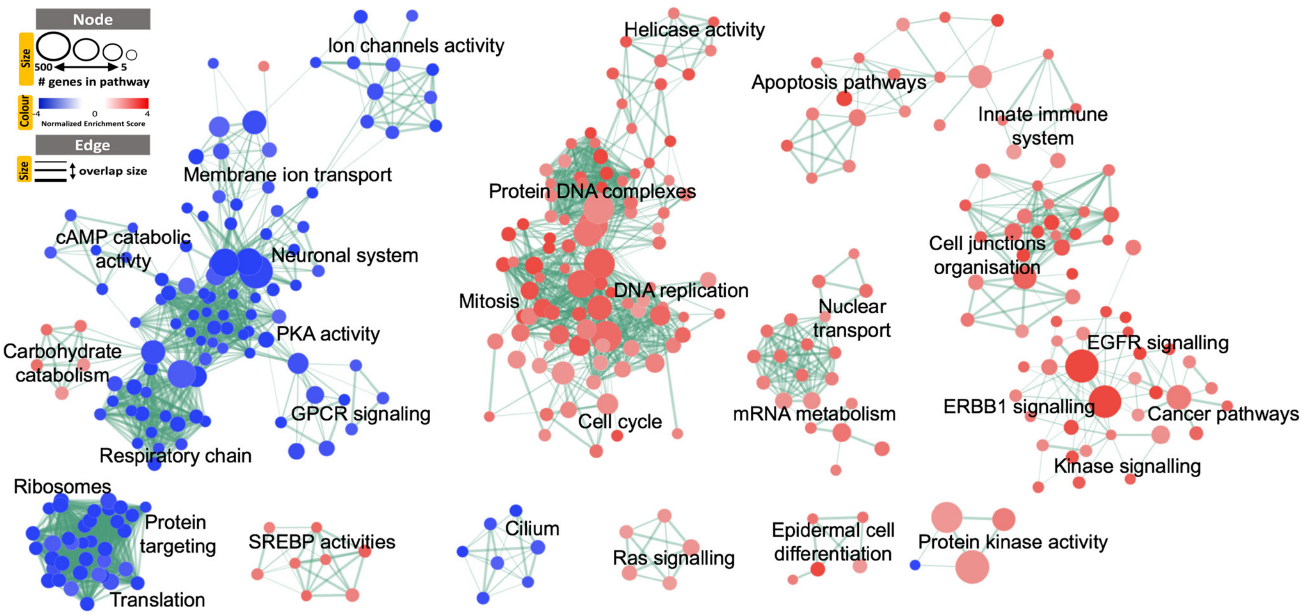

Supplementary Figure 2: Enrichment map of classical vs. exocrine-like tumours: visualisation of enriched GO-terms obtained from GSEA results of classical vs. exocrine-like tumours. Each node represents a GO-term with similar nodes clustered together and connected by edges whereby the number of known interactors between the nodes specifies the edge thickness. The size of each node denotes the gene set size for each specific node GO-term. The map was created the Enrichment Map plug-in for Cytoscape.

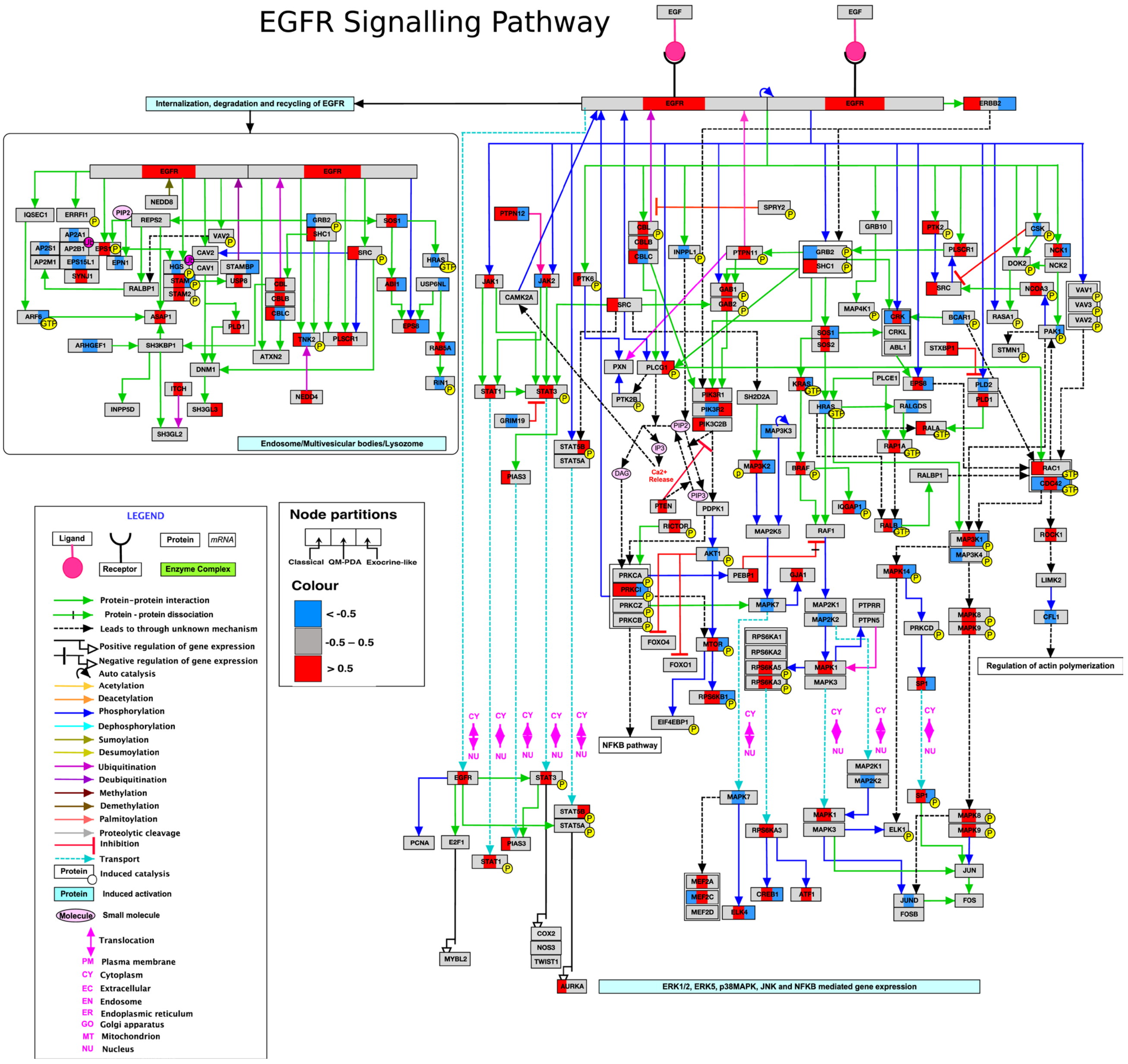

Supplementary Figure 3: Subtype-specific EGFR signaling pathway activity: a comparison of EGFR pathway activity between pancreatic cancer subtype based on mRNA expression data. Node denote genes—left section = classical, middle section = QM-PDA and right section = exocrine-like tumours. Node are coloured based on overall subtype mRNA-expression z-score (blue = low, grey = no change, and red = high). Edges represent various types of protein interaction (refer to legend to full notations for all edges).

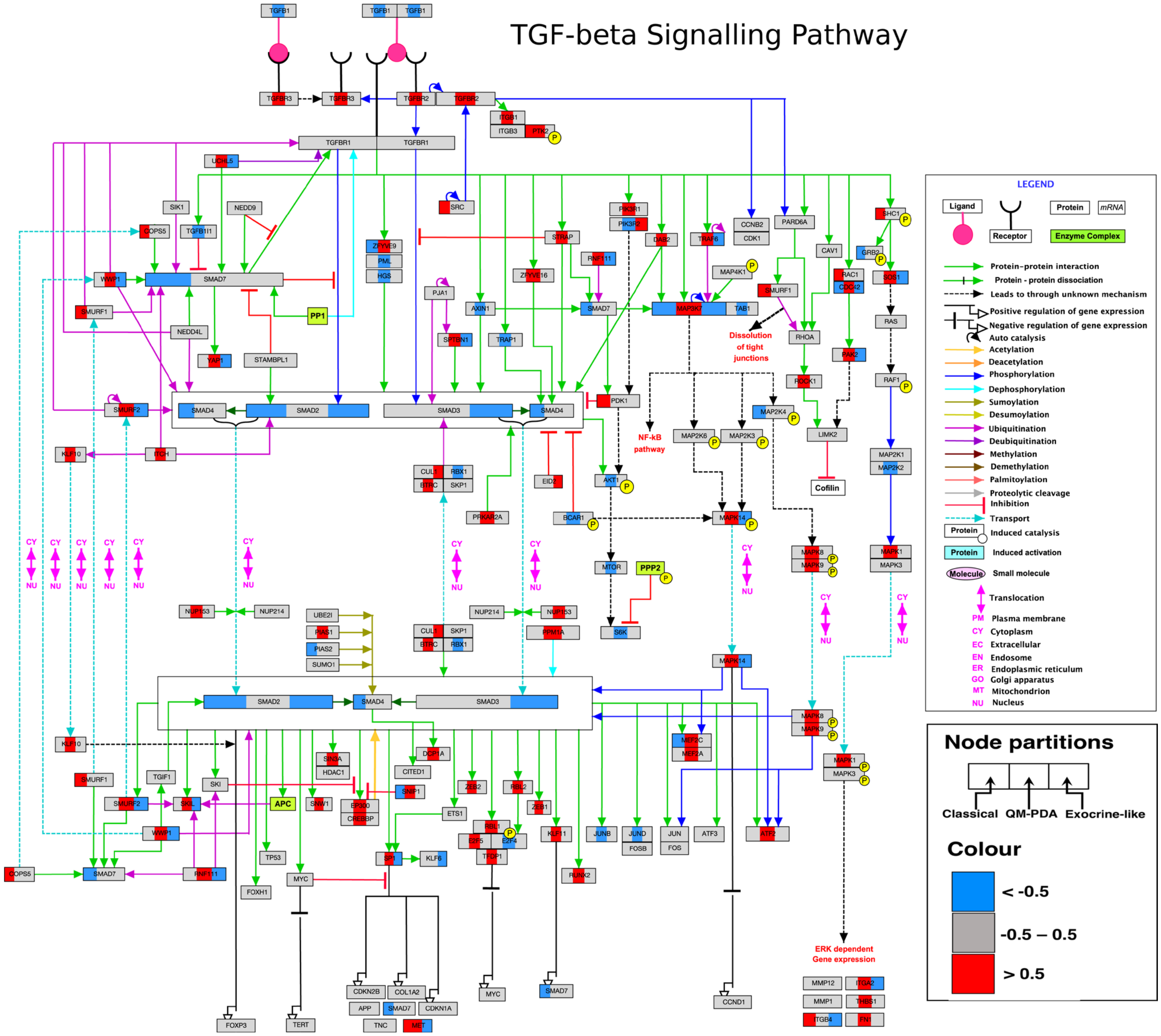

Supplementary Figure 4: Subtype-specific TGF-β signaling pathway activity: a comparison of TGF-β pathway activity between pancreatic cancer subtype based on mRNA expression data. Node denote genes; left section = classical, middle section = QM-PDA and right section = exocrine-like tumours. Node are coloured based on overall subtype mRNA-expression z-score (blue = low, grey = no change, and red = high). Edges represent various types of protein interaction (refer to legend to full notations for all edges).

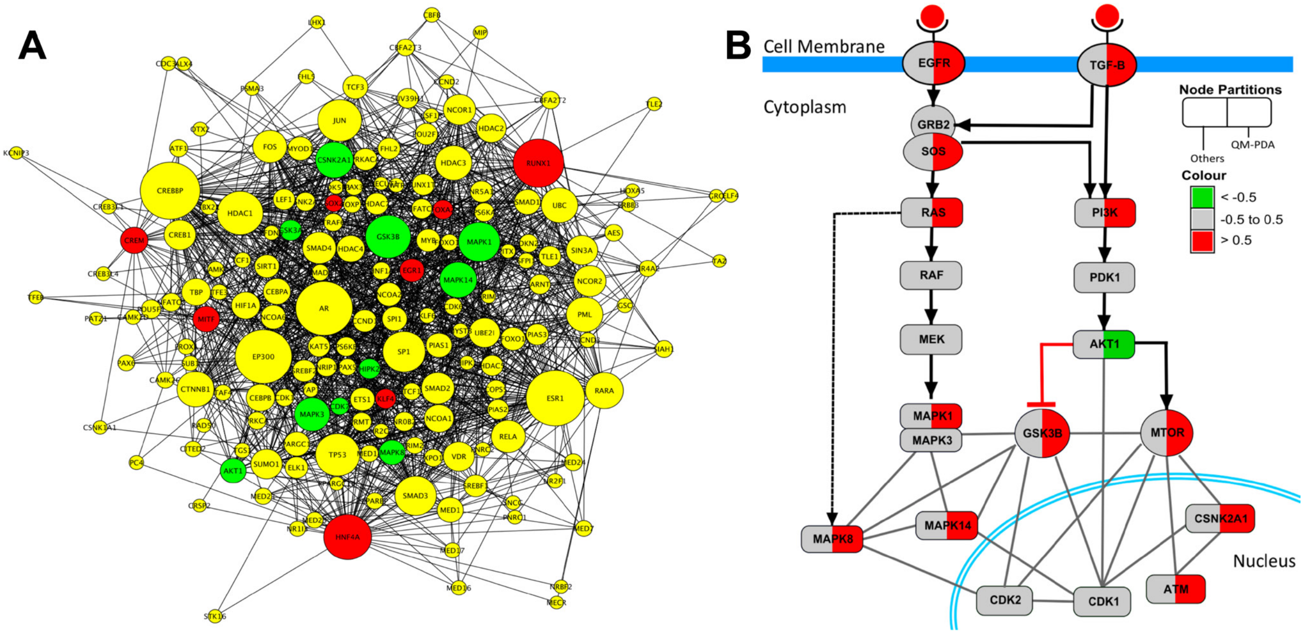

Supplementary Figure 5: (**A**) Intermediate protein-protein interaction subnetwork created during X2K analysis: the sub-network has 180 nodes with 1816 edges and is enriched in co-regulators, kinases and TFs that are experimentally verified to physically interact. (**B**) Simplified EGFR and TFG-β signaling pathway for X2K: mapping of mRNA expression data onto a model pathway showing the top ten X2K predicated kinase; AKT1, GSKB, MTOR, MAPK1, MAPK14, MAPK7, CDK1, CDK2 and ATM (also see Figure 2C). Each node denotes a pathway protein, the left section denotes QM-PDA z-scored mRNA expression, and the right section denotes Other subtypes z-scored mRNA expression. Green = low expression, grey = no change, and red = high expression.

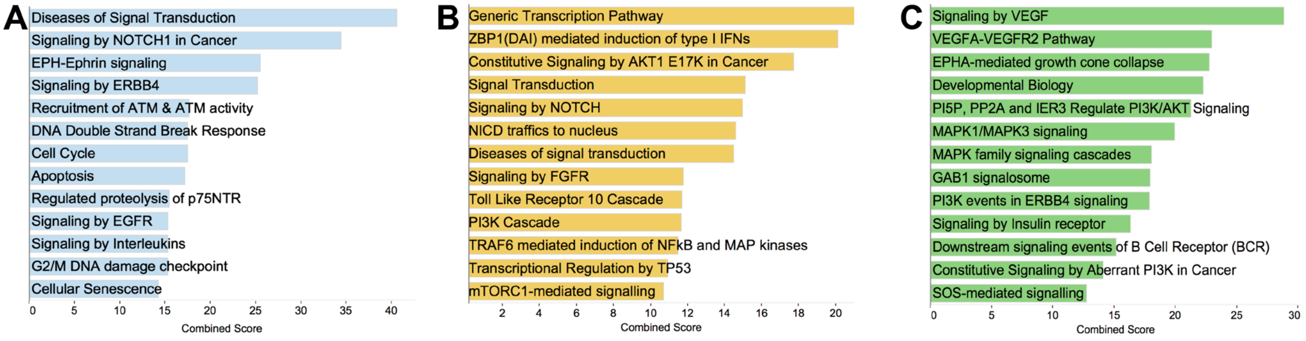

Supplementary Figure 6: Enriched Reactome pathways: Showing the top ranked dysregulated Reactome pathways based on mutations that are specific to each pancreatic cancer subtype. The size of each bar represents the combined score for each pathway (also see Supplementary File 5). (**A**) Top 13 pathways over presented by mutations unique to classical tumours. (**B**) Top 13 pathways over presented by mutations unique to QM-PDA. (**C**) Top 13 pathways over presented by mutations unique to exocrine-like tumours.

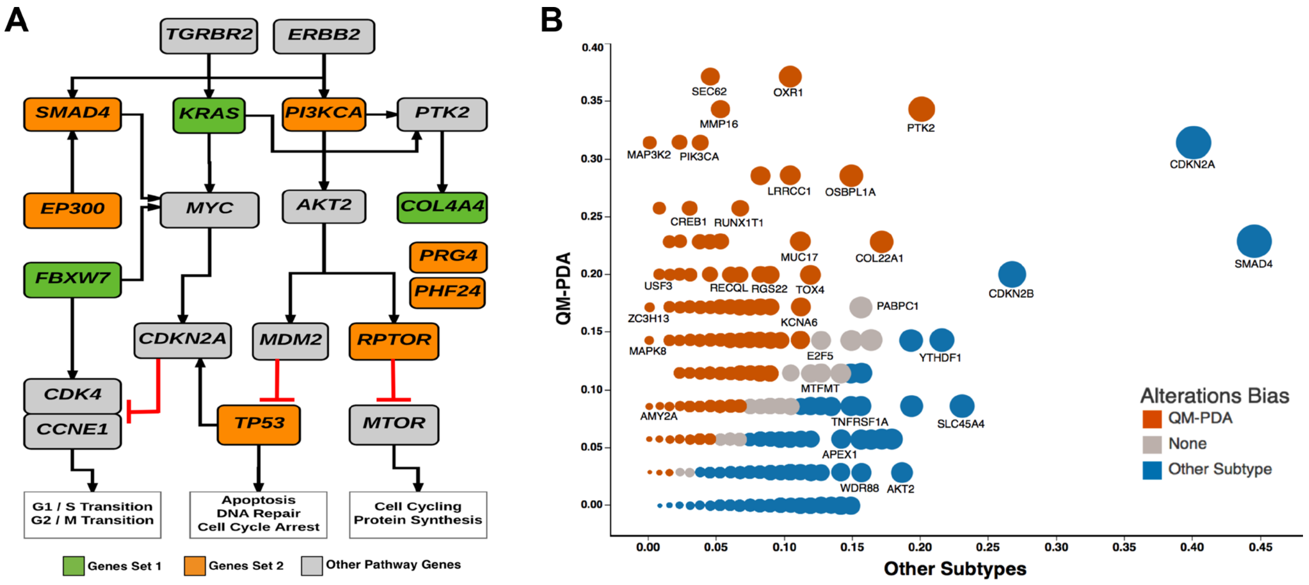

Supplementary Figure 7: (**A**) CoMDP Driver Pathway: An expanded pathway solution for the CoMDP test with $K = 10$. Node are colour represent gene set pathway class: green and brown nodes belong co-occurring driver pathways solutions one and two, whereas grey nodes represents connecting nodes. Network edges were curated from literature and various databases of protein-protein interactions. (**B**) Integrated alterations in pancreatic cancer subtypes: Shows alterations in QM-PDA vs. Other Subtypes. Each node represents a gene, who's colour shows details about the bias alterations and the node size is proportional to the overall alteration frequency in in pancreatic cancer. *TP53* and *KRAS* are excluded from the plot and they don't show any bias.

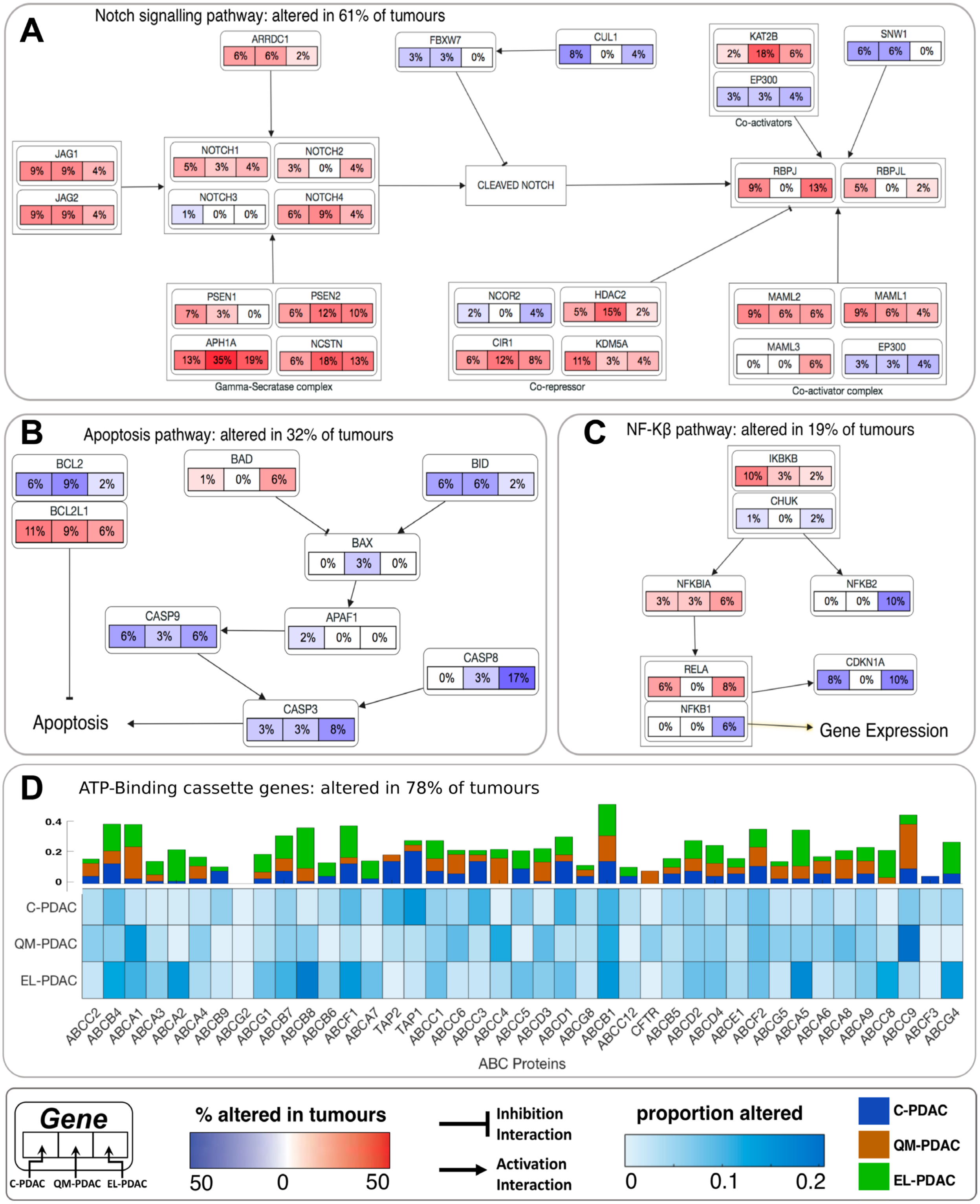

Supplementary Figure 8: Genetic alterations in three critical signaling pathways: Integrated alterations of mutations, copy number alteration, mRNA expression and protein levels are shown for (**A**) Notch signaling pathway (**B**) apoptosis pathway, and (**C**) NF-kβ pathway. Red indicates activating genetic alterations whereas blue indicates inactivating alterations. Darker shades correspond to higher alteration frequencies. Each node within the pathway represent a gene and the highlighted segments within each node and the percentage representing the alteration in the three PDAC subtype: C-PDAC, QM-PDAC and EL-PDAC from left, centre, and right, respectively. (**D**) The heatmap of integrated alterations in ATP-binding cassette encoding genes. The intensity denotes the frequency of alterations, with darker shades of blue representing a higher proportion of alterations.

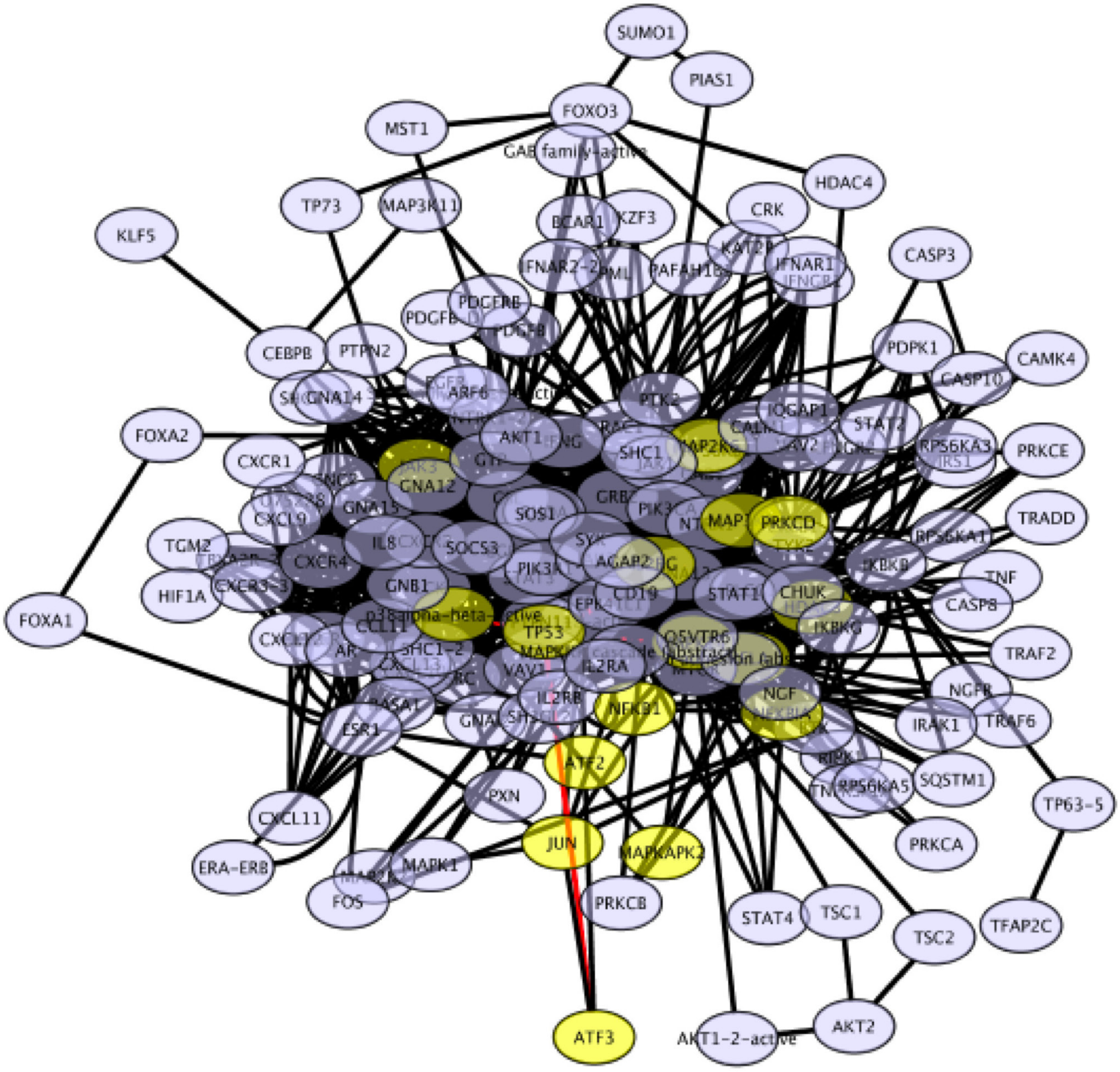

Supplementary Figure 9: TieDIE solution network: Visualisation of the TieDIE solution gene connectivity subnetwork. The sub-network has 158 nodes with 1127 edges and is enriched in mutated proteins and their TF targets likely response for the molecular differences between QM-PDA and other pancreatic cancer subtypes. The nodes in highlighted in yellow represent the *TP53* heat-diffusion network extracted the TieDIE solution network.

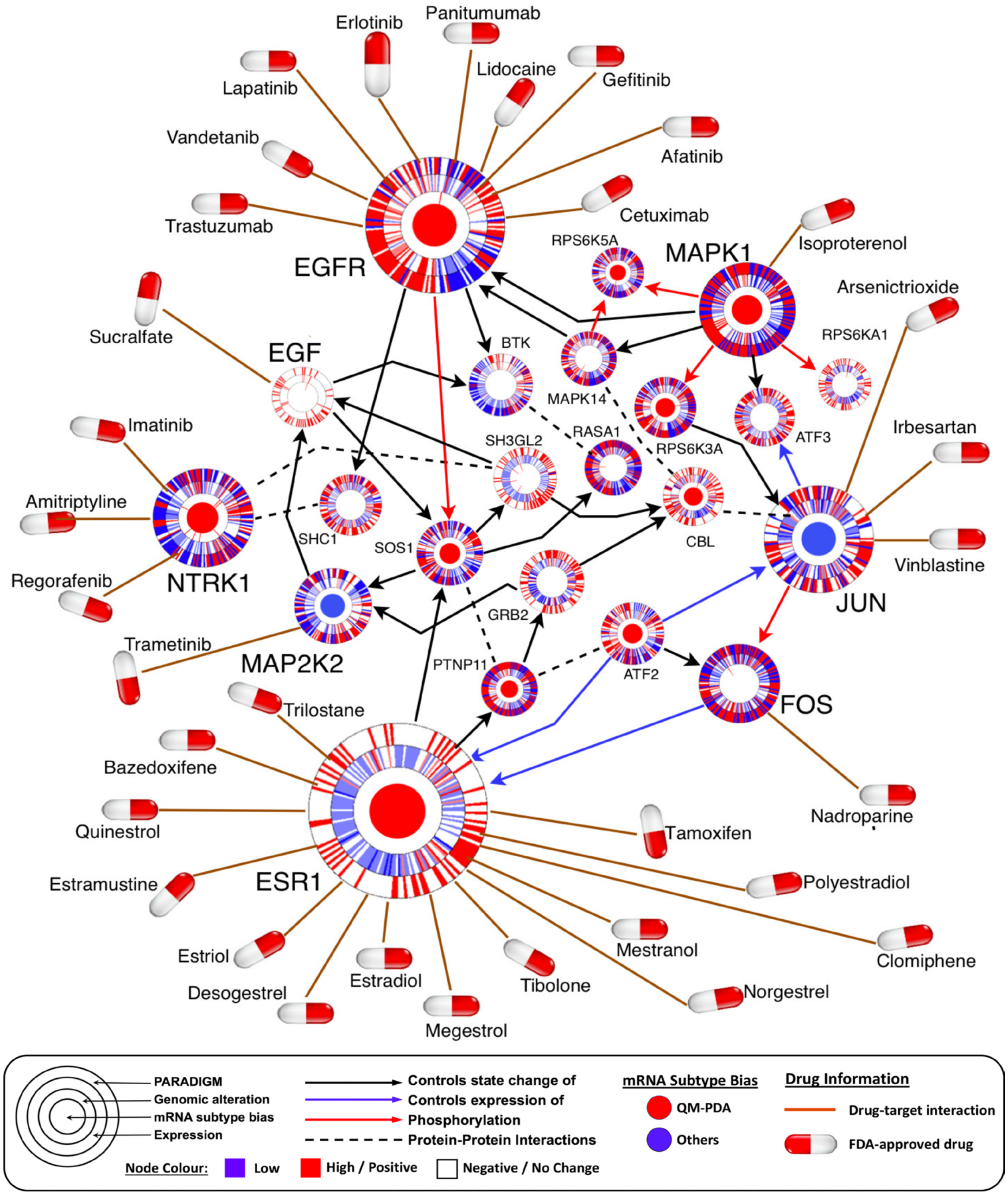

Supplementary Figure 10: MAPK heat diffusion sub-network: pathway extracted from the TieDIE solution network by heat diffusion for the MAPK1 network node and annotated with FDA approved anticancer drugs that are known to target specific pathway proteins. Each node indicates a pathway protein shown as concentric rings. The size of the node is proportional to the number of FDA approved drug for that particular pathway protein. The inner node detonates mRNA differential expression (Benjamini-Hochberg adjusted $p < 0.05$) biased for the statistically significant expressed gene between QM-PDA and other subtypes (red = QM-PDA bias, blue = bias towards other subtypes). The second ring shows the presence of genomic alterations for that gene in each sample that is denoted by a spoke within the ring. The third ring shows mRNA expression levels per samples (red = high, blue = low). The outer ring shows PARADIGM inferred activity for that gene in each sample (red = high, blue = low). Arrows indicate known protein-protein interaction extracted from UCSC Super pathway, KEA, ChEA or curated from the literature. We have made the visualisation easy by omitting some interactions between network nodes.

Supplementary File 1: Differentially expressed within the tumours of each PDAC subtypes. See Supplementary_File_1

Supplementary File 2A: Complete GSEA for C-PDAC vs QM-PDAC, C-PDAC vs EL-PDAC and QM-PDAC vs EL-PDAC, are Supplementary File 2A. See Supplementary_File_2A

Supplementary File 2B: Complete GSEA for C-PDAC vs QM-PDAC, C-PDAC vs EL-PDAC and QM-PDAC vs EL-PDAC, are Supplementary File 2B. See Supplementary_File_2B

Supplementary File 2C: Complete GSEA for C-PDAC vs QM-PDAC, C-PDAC vs EL-PDAC and QM-PDAC vs EL-PDAC, are Supplementary File 2C. See Supplementary_File_2C

Supplementary File 3: Expression 2Kinase of computationally predicted kinases and their rankings. See Supplementary_File_3

Supplementary File 4: List of mutations that are common among subtypes or unique to particular subtypes. See Supplementary_File_4

Supplementary File 5: Reactome pathways affected by genomic alterations across PDAC subtypes. See Supplementary_File_5

Supplementary File 6: PARADIGM predicted integrated pathway activity of all protein in all samples. See Supplementary_File_6
